# Comparison of the Protective Efficacy of Neutralizing Epitopes of 2009 Pandemic H1N1 Influenza Hemagglutinin

**DOI:** 10.3389/fimmu.2017.01070

**Published:** 2017-08-31

**Authors:** Bo Peng, Na Peng, Yanan Zhang, Fenghua Zhang, Xuguang Li, Haiyan Chang, Fang Fang, Fuyan Wang, Fangguo Lu, Ze Chen

**Affiliations:** ^1^College of Life Science, Hunan Normal University, Changsha, China; ^2^Centre for Biologics Evaluation, Biologics and Genetic Therapies Directorate, Health Products and Food Branch, Health Canada, Ottawa, ON, Canada; ^3^Department of Immunology, Xiangya School of Medicine, Central South University, Changsha, China; ^4^School of Medicine, Hunan University of Chinese Medicine, Changsha, China; ^5^Shanghai Institute of Biological Products, Shanghai, China

**Keywords:** 2009 pandemic H1N1 influenza, hemagglutinin, epitope, plasmid DNA, protective efficacy

## Abstract

The 2009 H1N1 influenza (Pdm09) pandemic has been referred to as the first influenza pandemic of the twenty-first century. There is a marked difference in antigenicity between the pandemic H1N1 virus and past seasonal H1N1 viruses, which allowed the pandemic virus to spread rapidly in humans. Antibodies (Abs) against hemagglutinin (HA), especially neutralizing Abs against epitopes in the head of HA, play critical roles in defending the host against the virus. Some preexisting neutralizing Abs that recognize neutralizing epitopes of Pdm09 HA, thereby affording cross-protection, have been reported. To better understand the protective effects of epitopes in Pdm09 HA, we constructed a series of plasmid DNAs (DNA vaccines) by cloning various combinations of Pdm09 neutralizing epitopes into the HA backbone derived from A/PR/8/1934 (H1N1). We subsequently compared the protective immune responses induced by these various forms of HA in a mouse model. We found that the plasmid DNAs with epitope substitutions provided better protection against lethal virus challenge and induced higher strain-specific antibody titers, with epitope Sa being the most effective. Moreover, the combination of epitopes Sa and Sb provided almost complete protection in mice. These findings provide new insights into the protective efficacy of neutralizing epitopes of influenza HA.

## Introduction

Influenza presents a significant negative impact on public health. Both seasonal influenza epidemics and occasional outbreaks of global pandemics can result in high morbidity and mortality rates (1). In 2009, a new influenza pandemic emerged and quickly spread to 214 countries (2). The pathogen of this first influenza pandemic in the twenty-first century was identified as a novel influenza A virus (Pdm09), a triple-reassortant of human, bird, and swine influenza viruses (3). The rapid transmission of this virus in humans resulted from its remarkably different genetic make-up and its antigenicity from past seasonal influenza viruses (4, 5).

The entry of the virus to the host cells is mediated by the major envelope glycoprotein hemagglutinin (HA), a surface viral protein and primary target of protective humoral immune responses (6). The protection elicited by virus infection or vaccination is largely mediated through neutralizing antibodies (Abs) targeting the globular head of HA (6–9). The HA of the H1 subtype A/PR/8/34 (PR8) contains five neutralizing epitopes previously identified by Gerhard and colleagues using a large panel of monoclonal Abs; these epitopes were designated Sa, Sb, Ca1, Ca2, and Cb (10, 11).

An epidemiological survey showed that Pdm09 was more infectious in young people than in the elderly (12). The lower infection rate of the elderly (>65 years) likely reflects the presence of highly cross-reactive protective Abs elicited by previous infection (5). Subsequent studies in mouse model demonstrated that Pdm09 and the 1918-like strains share marked similarities in their antigenicity but substantially differed from recently circulating H1 strains. Vaccination of mice with viruses from previous years could afford protection against lethal Pdm09 virus challenge. Moreover, further epitope analysis revealed that the epitope Sa played an important role in inducing cross-protective immune responses (13).

In the present study, we addressed two questions: could the other four neutralizing epitopes confer protection against the pandemic virus infection? Is there any difference between these epitopes with respect to protective efficacy? To this end, we constructed a series of plasmid DNAs (DNA vaccines) by replacing one or two epitopes of the PR8 HA with epitopes derived from the Pdm09 HA. Subsequently, we immunized mice with these mutant plasmid DNAs, followed by lethal viral challenge using mouse-adapted Pdm09 virus. The results showed that plasmid DNAs with single-epitope substitutions provided partial but better protection than plasmid DNAs with the original PR8 backbone. Furthermore, the substitution of two epitopes greatly enhanced protection. Importantly, DNA constructs with substitutions of both Sa and Sb epitopes afforded the best protection, with 100% of animals surviving the lethal virus challenge.

## Materials and Methods

### Animals

Specific pathogen-free (SPF) female BALB/c mice (6–8 weeks old) were purchased from the Center for Disease Control and Prevention of Hubei Province and were housed under SPF conditions with constant temperature and humidity. All mice had free access to food and water. All experiments with mice were reviewed and approved by the Institutional Animal Care and Use Committee of Hunan Normal University. All animal procedures were performed in accordance with the animal ethics guidelines of the Chinese National Health and Medical Research Council.

### Plasmid DNAs

Plasmids pCAGGSP7/Pdm09 HA (pPdm09 for short) and pCAGGSP7/PR8 HA (pPR8 for short) were constructed by inserting the full-length HA gene from the corresponding influenza virus into the eukaryotic expression vector pCAGGSP7 (14, 15).

**Figure 1:**
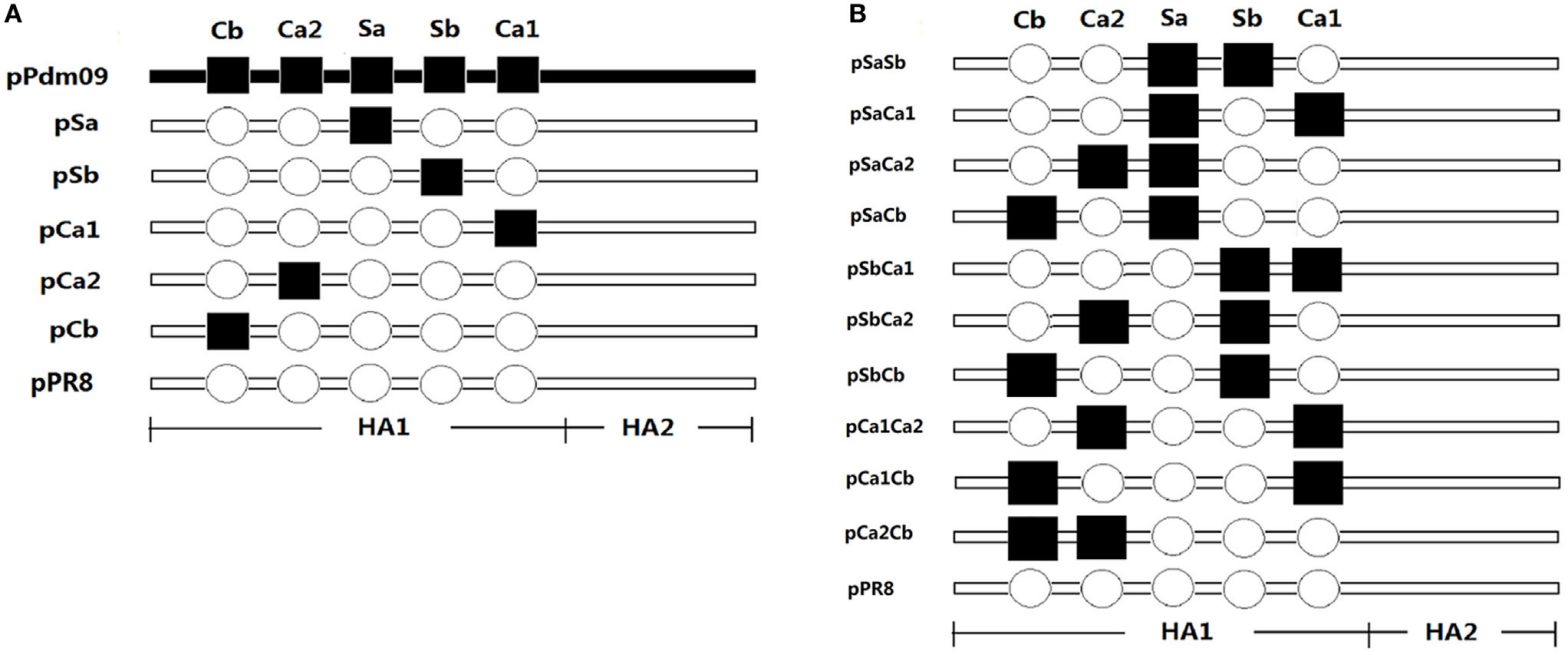
Schematic diagram of the plasmid DNAs. Plasmids pCAGGSP7/Pdm09 HA (pPdm09) and pCAGGSP7/PR8 HA (pPR8) were constructed by inserting full-length hemagglutinin (HA) gene from the Pdm09 and PR8 influenza virus, respectively, into the eukaryotic expression vector pCAGGSP7. Hollow circles denote epitopes of PR8 HA, while filled squares denote corresponding epitopes of Pdm09 HA. Plasmid pPR8 was chosen as a recipient HA backbone and DNA fragments containing epitopes of the Pdm09 HA were synthesized to replace of the corresponding epitope sequence in pPR8. **(A)** Plasmid DNAs substituted with one epitope of the Pdm09 HA was constructed and denoted pSa, pSb, pCa1, pCa2, and pCb. **(B)** Plasmid DNAs substituted with two epitopes of the Pdm09 HA were constructed and denoted pSaSb, pSaCa1, pSaCa2, pSaCb, pSbCa1, pSbCa2, pSbCb, pCa1Ca2, pCa1Cb, and pCa2Cb.

DNA fragments containing epitope Sa of the Pdm09 HA were synthesized (Sangon Biotech, Shanghai, China) and used to replace the corresponding epitope sequence of pPR8 to generate a new plasmid DNA pCAGGSP7/PR8 HA-Pdm09-Sa (pSa). Using the same procedure, we then constructed four other plasmid DNAs substituted with one epitope of the Pdm09 HA, denoted as pSb, pCa1, pCa2, and pCb (Figure 1A). There were altogether 10 combinations for plasmid DNAs substituted with two epitopes of the Pdm09 HA. These plasmids were also produced as above and individually denoted as pSaSb, pSaCa1, pSaCa2, pSaCb, pSbCa1, pSbCa2, pSbCb, pCa1Ca2, pCa1Cb, and pCa2Cb (Figure 1B).

The plasmids were propagated in *Escherichia coli* XL1-blue strain and were purified using the NucleoBondXtra Maxi purification kit (Macherey-Nagel, Germany).

### Virus and Cells

The mouse-adapted influenza strain NYMC X-179A (H1N1), which contains Pdm09 HA, was produced by serial lung-to-lung passage in mice as described in our previous studies (14, 16). The virus was aliquoted and stored at −80°C until use. This virus was lethal for mice and a 50% mouse lethal dose (MLD_50_) of each stock was determined.

Madin-Darby canine kidney (MDCK) cells were maintained in cell growth medium (DMEM containing 100 U/ml penicillin, 100 µg/ml streptomycin, and 10% heat inactivated fetal calf serum) at 37°C in a 5% CO_2_ humidified atmosphere.

### Immunization and Challenge

Twenty-six mice per group were immunized by electroporation as previously described (17–20). After the injection of mice in the quadriceps with 30 µg of plasmid DNA dissolved in 40 µl of Tris-EDTA buffer, electric pulses were immediately delivered using an electric pulse generator (ECM830, BTX, CA). Three 100-volt pulses were delivered for 50 ms at a rate of one pulse per second, followed by three pulses of the opposite polarity. A boost immunization was administered 3 weeks later. Unimmunized mice were used as controls.

Two weeks after the last immunization, all mice were anesthetized and intranasally challenged with 20 µl of viral suspension containing 20 × MLD_50_ mouse-adapted virus. Twenty mice per group were randomly chosen to be monitored daily for survival and body weight for 14 days. The remaining six mice per group were sacrificed to collect bronchoalveolar fluid (BAL) at 4 days after the challenge.

### Detection of Abs

One day before the challenge, blood was collected from the caudal vein. The titers of serum anti-HA IgG Abs were determined using an enzyme-linked immunosorbent assay (ELISA) as previously described (21). Briefly, 96-well microtiter plates (Costar, MA, USA) were coated with inactivated NYMC X-179A vaccine (Shanghai Institute of Biological Products, Shanghai, China) and blocked with 1% bull serum albumin in PBS overnight. Serial dilutions (twofold) of sera from each group of mice were added, followed by biotinylated goat anti-mouse IgG (catalog number1030-08, Southern Biotechnology Associates, AL, USA) and subsequently streptavidin conjugated with alkaline phosphatase. The plates were finally developed with the substrate *p*-nitrophenyl-phosphate, and the amount of chromogen produced was measured at 450 and 405 nm using an ELISA reader (Labsystems Multiskan Ascent Autoreader, Helsinki, Finland). Positive cutoff values were set as the means + 2 × SD of unimmunized sera. The ELISA Ab titer was expressed as the reciprocal of the highest serum dilution.

### Hemagglutination-Inhibition (HI) Assay

The HI assay was performed according to the WHO protocol (22). Briefly, sera were treated with receptor-destroying enzyme (RDE) at 37°C for 20 h and subsequently inactivated at 56°C for 30 min. The inactivated sera were subsequently serially diluted (twofold) and added in duplicates to V-shaped 96-well plate with 25 µl in each well. Next, 25 µl of viral antigen containing four hemagglutinating units was added to each well, and the plates were incubated at room temperature for 15 min, followed by the addition of 50 µl of 0.5% chicken red blood cells. The plate was read for complete hemagglutination after incubation at room temperature for 30 min. The HI titers were determined as the highest serum dilution showing hemagglutination inhibition.

### Virus Micro-Neutralization (VN) Assay

The VN assay was carried out as described by Cox et al. (23). Briefly, MDCK cells were seeded onto 96-well plates and washed with serum-free DMEM. Sera treated with RDE were serially diluted (twofold) with a starting dilution of 1:10 and subsequently mixed with 100 × TCID_50_ of NYMC X-179A virus for 1 h before adding to the MDCK cells. After incubation with the cells for 1 h, the virus–serum mixture was replaced with 200 µl of viral growth medium (serum-free complete DMEM supplemented with 2 µg/ml of TPCK-treated trypsin). The cells were incubated for an additional 72 h, and virus activity was determined based on hemagglutination. The VN titers were determined as the highest serum dilution displaying viral inhibition.

### Virus Titrations

Virus titration was performed as previously described (24). BAL was serially diluted (10-fold) to inoculate MDCK cells, which were subsequently incubated in the growth medium and tested for hemagglutination after 72 h. The lung virus titer in each sample, expressed as the TCID_50_, was calculated according to Reed and Muench method (25).

### Statistical Analyses

Comparisons of the experimental groups were performed using one-way ANOVA (GraphPad Prism); for survival rate, the probability was calculated using Fisher’s exact test. The difference was considered significant when the *p*-value was less than 0.05.

## Results

### Construction of Plasmid DNAs

Both PR8 and Pdm09 viruses belong to the H1 subtype. Although amino acid sequence analysis of the Pdm09 HA and the PR8 HA showed some mutations in their neutralizing epitopes (Table 1), crystal structure analysis revealed that the overall structures of the HA proteins were almost identical (26, 27). Therefore, we hypothesized that the neutralizing epitopes could be swapped from one strain to the other, resulting in new forms of HA capable of inducing protective immune responses in animals. To this end, plasmid pCAGGSP7/PR8 HA (pPR8), expressing the full-length HA of PR8, was selected as the backbone (recipient) and designated the unmodified “negative” control in the experiments. DNA fragments containing one or two epitopes of the Pdm09 HA were obtained through gene synthesis, and a series of plasmid DNAs substituted with one or two epitopes derived from the Pdm09 HA were constructed by substituting the corresponding epitope sequence in the pPR8 (backbone). The plasmid pCAGGSP7/Pdm09 HA (pPdm09 for short), expressing full-length HA of Pdm09 virus, was set as the unmodified “positive” control. All plasmid constructions were confirmed through DNA sequencing.

**Table 1:** Comparison of amino acid sequence of neutralizing epitopes of the Pdm09 HA and PR8 HA (H1 numbering).

Virus strain	Cb	Ca2	Sa	Sb	Ca1

	87–92	153–158 237–238	141–142 169–173 175–180	200–211	182–186 219–221 251–253
Pdm09	LSTASS	PHAGAK RD	PN KKGNS PKLSKS	TSADQQSLYQNA	INDKG SSR EPG
PR8	L**LPVR**S	**S**H**E**G**KS** RD	PN **E**K**EG**S PKL**KN**S	**N**S**KE**QQ**N**LYQN**E**	**V**N**K**KG **T**S**NK**PG

*Variations of amino acids are labeled in bold and underlined*.

### Comparison of the Protective Efficacy of Plasmid DNAs Substituted with One Epitope of the Pdm09 HA

The expression of the Cb, Ca2, Sa, Sb, and Ca1 epitopes was analyzed using immunofluorescence images, western blotting, and haemadsorption analyses. To this end, 293T cells were transfected with plasmids encoding proteins with each of the epitopes. As shown in Figures S1–S3 in Supplementary Material, both the expression and proper folding of the epitopes were confirmed.

To compare the ability of various plasmid DNAs to induce protective immune responses, BALB/c mice were randomly divided into 8 groups with 26 mice per group. Seven experimental groups were immunized twice through intramuscular electroporation at an interval of 3 weeks; one group of unimmunized mice was used as the control. Two weeks after the boosting, all mice were challenged with a lethal dose of mouse-adapted Pdm09 virus.

**Figure 2:**
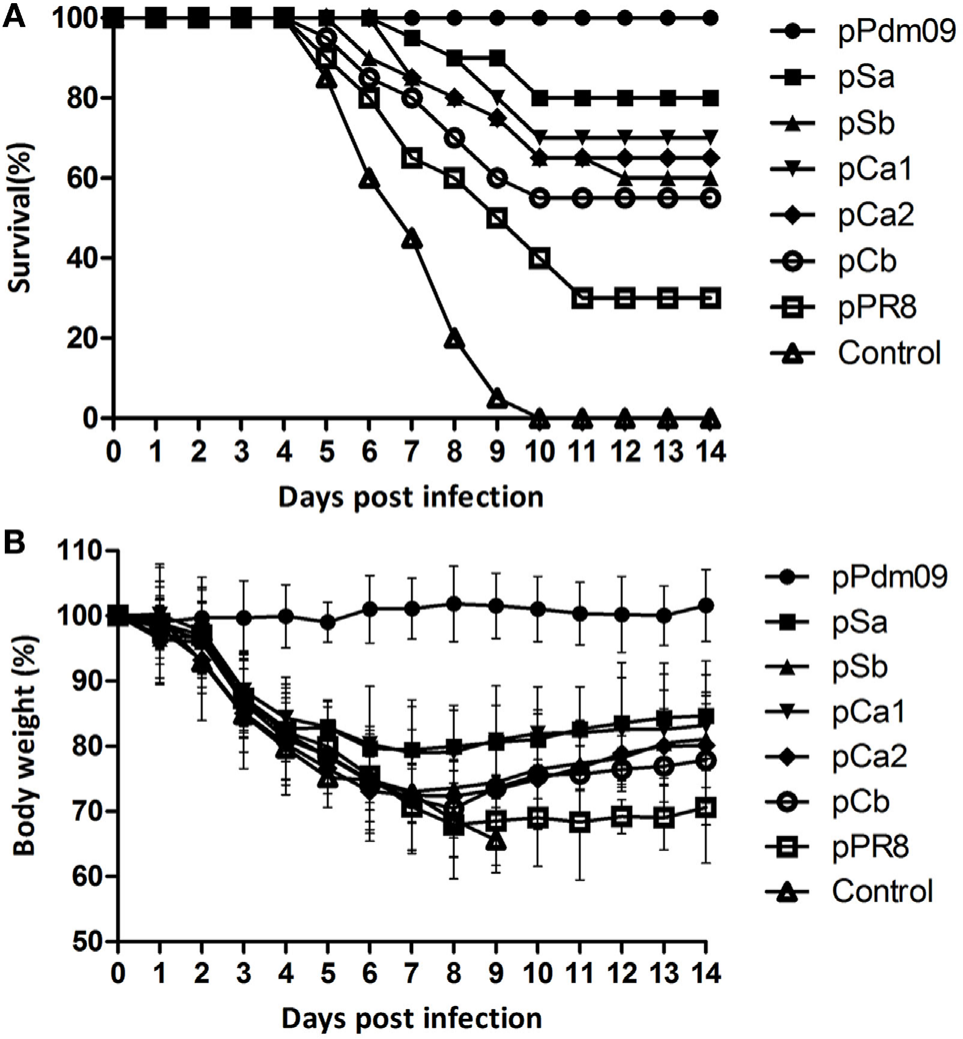
Survival and body weight of mice immunized with plasmid DNAs substituted with one epitope of the Pdm09 HA. Female BALB/c mice (6–8 weeks old) were immunized twice, at an interval of 3 weeks, with 30 µg of plasmid DNAs substituted with one epitope of the Pdm09 HA. Two weeks after the boost, the mice were challenged with a lethal dose (20 × MLD_50_) of mouse-adapted Pdm09 virus and were monitored for survival and body weight for 14 days, **(A)** Survival, **(B)** body weight; error bars show SD of each group.

As expected, on day 3, the control mice initially presented severe clinical symptoms such as hunched posture, weakness, ruffled fur, and substantial weight loss (Figure 2). Robust viral replication following viral challenge was detected in the lungs of unimmunized animals, and all animals succumbed to death within 10 days post-challenge (Table 2). In stark contrast, mice immunized with pPdm09 were completely protected from challenge. Moreover, mice immunized with pPR8 showed 30% survival rates and approximately 32% weight loss. Remarkably, the survival rates of mice receiving plasmid DNAs containing one epitope of the Pdm09 HA were drastically improved; specifically, a significantly higher survival rate was observed in mice immunized with pSa or pCa1, with survival rates of 80 and 70%, respectively. Other single substitutions, i.e., pSb, pCa2, or pCb, also provided better protection than DNA vaccines with no substitutions.

**Table 2:** Protection against lethal virus challenge of mice immunized with plasmid DNAs substituted with one epitope of the Pdm09 HA.^a^

Plasmid DNA	No. of survivors/no. tested (ratio %)	Body weight loss (% of original weight)^b^	Lung virus titers (log_10_TCID_50_/ml)^b^
pPdm09	20/20 (100)^c^	1.0 ± 3.1^c^	0.2 ± 0.4^c^
pSa	16/20 (80)^c^	20.6 ± 6.7^c^	5.8 ± 0.8^c^
pSb	12/20 (60)	27.0 ± 9.5	6.8 ± 1.0
pCa1	14/20 (70)^c^	21.1 ± 8.2^c^	6.3 ± 1.2
pCa2	13/20 (65)	27.7 ± 6.4	7.0 ± 0.9
pCb	11/20 (55)	29.6 ± 7.5	7.2 ± 1.2
pPR8	6/20 (30)	32.1 ± 8.3	8.0 ± 0.6
Control	0/20 (0)	34.4 ± 5.0	9.3 ± 1.0

*^a^Female BALB/c mice (6–8 weeks old) were immunized twice, at an interval of 3 weeks, with 30 µg of plasmid DNAs substituted with one epitope of the Pdm09 HA. 2 weeks after the boost, the mice were challenged with lethal dose (20 × MLD_50_) of mouse-adapted Pdm09 virus and monitored for body weight loss and survival for 14 days (*n* = 20). The BAL was collected at 4 days post-infection for titration of lung virus (*n* = 6)*.

*^b^Results are expressed as the means ± SD in each group*.

*^c^Significant difference compared with pPR8 (*p* < 0.05)*.

To verify the data obtained in survival rate monitoring, we also determined the lung virus titers in mice at 4 days post viral challenge. Consistent with data for protection and weight loss, the control mice showed the highest viral titer in BAL (Table 2); nevertheless, pSa showed a significantly lower viral titer compared with the pPR8 group, and, as expected, no virus was detected in animals receiving the homologous Pdm09 vaccine.

Collectively, these results suggested that substitution of one epitope in PR8 HA with an epitope derived from a different Pdm09 HA could provide partial protection against lethal Pdm09 influenza infection, and the magnitude of protection in descending order was Sa, Ca1, Ca2, Sb, and Cb.

### Analyses of Antibody Responses in Mice Immunized with Plasmid DNAs Substituted with One Epitope of the Pdm09 HA

We next investigated the antibody responses elicited by these plasmid DNAs. To this end, 1 day before virus challenge, we examined serum samples for pandemic strain-specific immunoglobulin G (IgG), and subclasses IgG1 and IgG2a using ELISA. In parallel, hemagglutination inhibition and neutralizing Abs against the Pdm09 HA were also determined using HI and VN assays.

**Figure 3:**
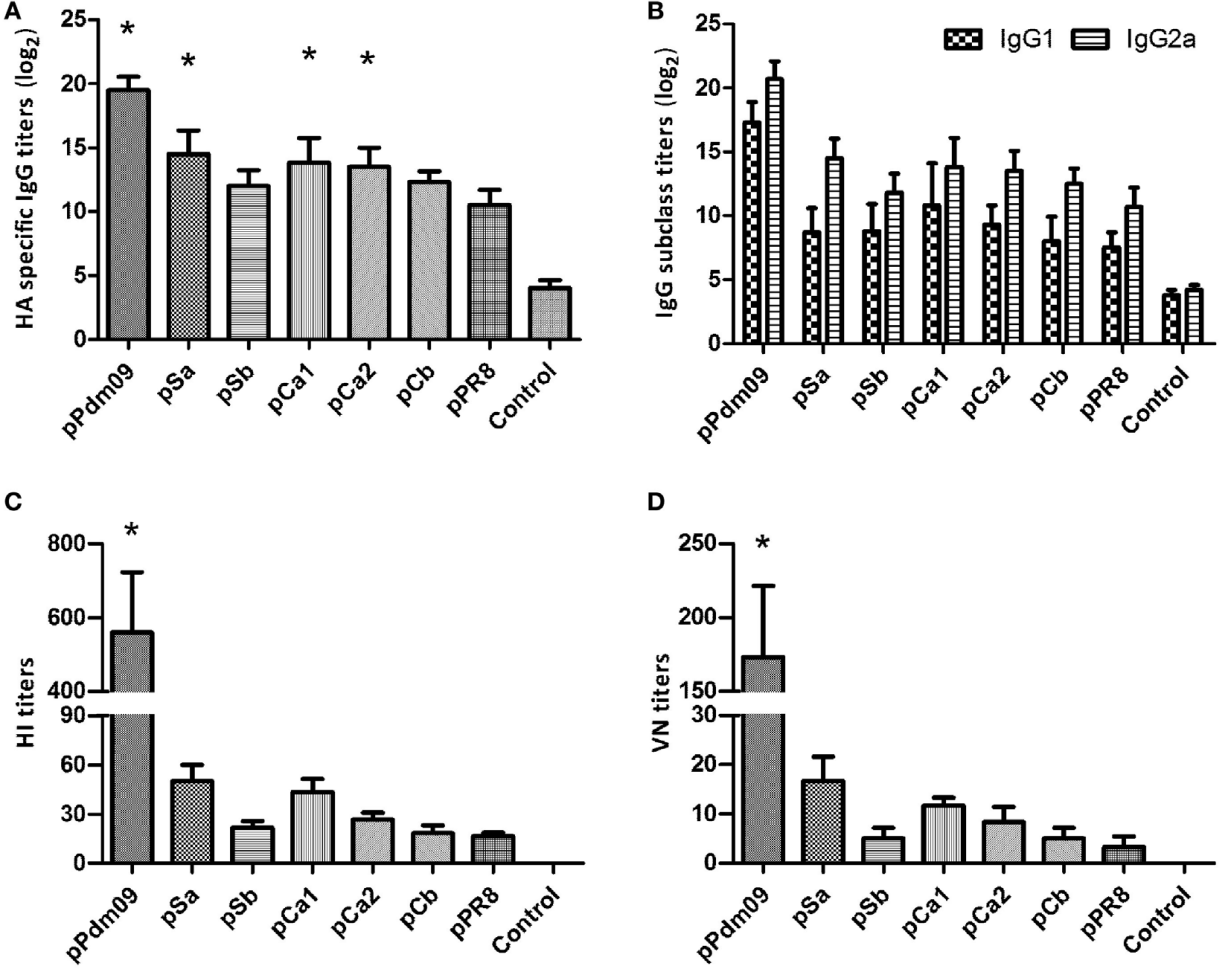
Antibody responses in mice immunized with plasmid DNAs substituted with one epitope of the Pdm09 HA. Female BALB/c mice (6–8 weeks old) were immunized twice, at an interval of 3 weeks, with 30 µg of plasmid DNAs substituted with one epitope of the Pdm09 HA. Thirteen days after the boost, the sera were collected for Ab analyses: **(A)** for immunoglobulin G (IgG); **(B)** for IgG subclass; **(C)** for hemagglutination-inhibition assay; and **(D)** for VN assay. Bars and whiskers represent the average and SD. Asterisk symbol (*) indicates a significant difference compared with pPR8 (*p* < 0.05).

As expected, the pPdm09 induced a significantly higher IgG antibody titer with increased HA inhibition and neutralizing activities (Figure 3). Notably, all plasmid DNAs containing one epitope of the Pdm09 HA induced higher IgG titers than pPR8, particularly in the case of pSa, pCa1, or pCa2 (*p* < 0.05). With regard to IgG subclass, all plasmid DNAs induced a higher ratio of IgG2a over IgG1, suggesting Th1-skewed immune responses. Furthermore, although mice immunized with plasmid DNAs containing one epitope of the Pdm09 HA showed higher HI and VN titers than those immunized with pPR8, the statistical analyses revealed no significant difference, suggesting that the antibody levels detected using ELISA may also contain non-neutralizing and non-HI Abs.

Taken together, the substitution of a single epitope could induce higher levels of HA strain-specific Abs, and the antibody titers were largely consistent with the data from bodyweight/survival rate observations. As substitution of a single epitope could only partially protect the animal from lethal virus challenge, it was necessary to determine whether more than one epitope would afford better protection.

### Comparison of Protective Efficacy of Plasmid DNAs Substituted with Two Epitopes of the Pdm09 HA

**Figure 4:**
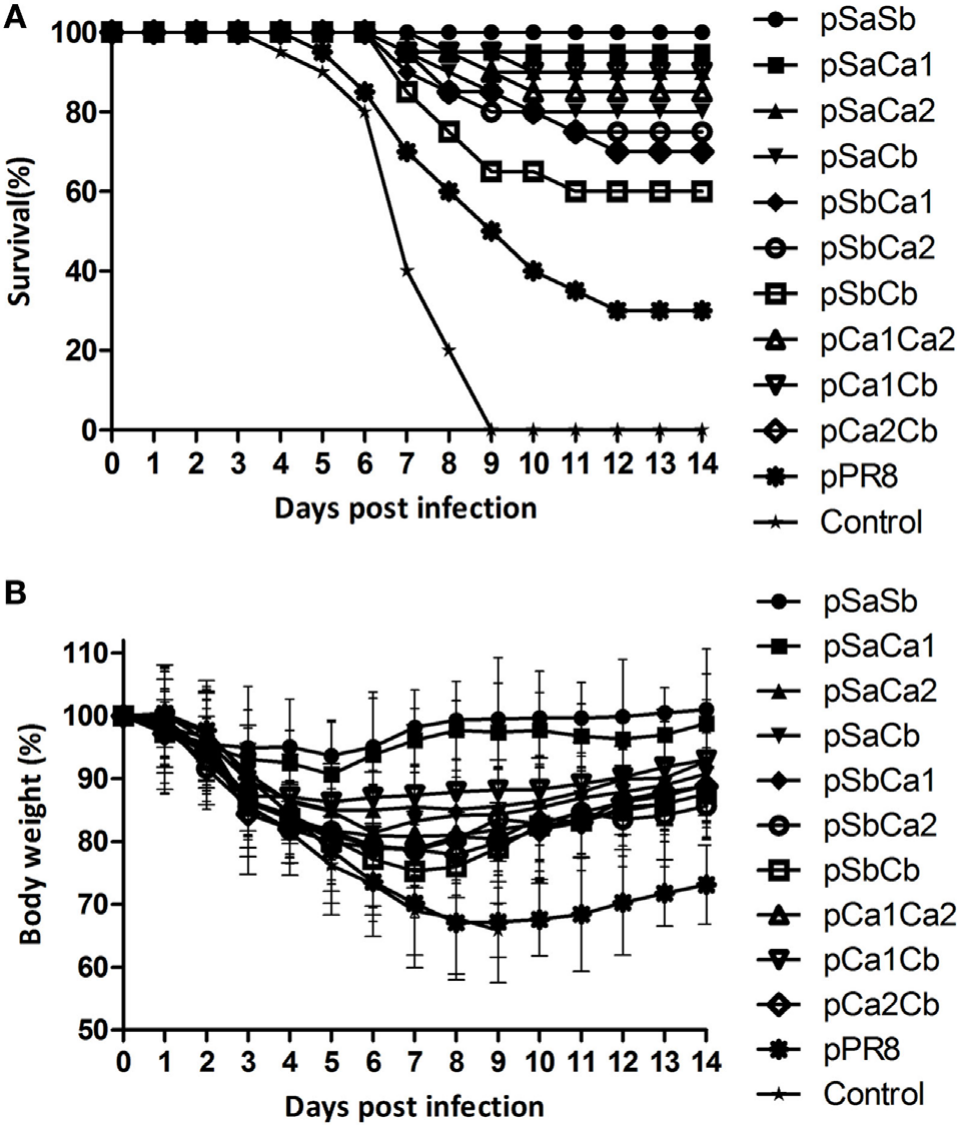
Survival and body weight of mice immunized with plasmid DNAs substituted with two epitopes of the Pdm09 HA. Female BALB/c mice (6–8 weeks old) were immunized twice, at an interval of 3 weeks, with 30 µg of plasmid DNAs substituted with two epitopes of the Pdm09 HA. Two weeks after the boost, mice were challenged with a lethal dose (20 × MLD_50_) of mouse-adapted Pdm09 virus and monitored for survival and body weight for 14 days. **(A)** For Survival and **(B)** for body weight, error bars show the SD of each group.

We next determined whether the replacement of two epitopes of the Pdm09 HA in PR8 HA could improve protection from lethal virus challenge with Pdm09 virus. To this end, mice were immunized with DNA plasmids containing two epitopes and were challenged as described above. As shown in Table 3, all groups, except pSbCb (survival rate 60%), induced significant higher survival rates (>70%) compared with the unmodified pPR8 group. Remarkably, pSaSb provided complete protection (survival rate 100%), and the mice in this group showed only 6.3% body weight loss and were quickly recovered from infection (Figure 4). The results of lung virus titration were also correlated with the data obtained from body weight and survival observations. Specifically, mice immunized with plasmid DNAs containing two epitopes showed lower lung virus titers, with both pSaSb and pSaCa1 being significantly lower than pPR8. These results indicate that the substitution of two epitopes can markedly enhance protective efficacy, with the combination of epitopes Sa and Sb affording the best protection against lethal virus challenge.

**Table 3:** Protection against lethal virus challenge of mice immunized with plasmid DNAs substituted with two epitopes of the Pdm09 HA.^a^

Plasmid DNA	No. of survivors/no. tested (ratio %)	Body weight loss (% of original weight)^b^	Lung virus titers (log_10_TCID_50_/ml)^b^
pSaSb	20/20 (100)^c^	6.3 ± 5.3^c^	3.3 ± 0.5^c^
pSaCa1	19/20 (95)^c^	9.3 ± 8.6^c^	5.0 ± 1.5^c^
pSaCa2	18/20 (90)^c^	15.0 ± 6.3^c^	5.3 ± 1.2
pSaCb	16/20 (80)^c^	18.6 ± 7.4^c^	6.0 ± 0.6
pSbCa1	14/20 (70)^c^	21.2 ± 9.5	6.5 ± 1.0
pSbCa2	15/20 (75)^c^	21.4 ± 5.6	6.7 ± 1.5
pSbCb	12/20 (60)	24.7 ± 7.4	7.3 ± 1.4
pCa1Ca2	17/20 (85)^c^	19.2 ± 8.1^c^	5.7 ± 2.3
pCa1Cb	18/20 (90)^c^	13.6 ± 11.2^c^	5.7 ± 1.8
pCa2Cb	14/20 (70)^c^	22.1 ± 6.8	6.8 ± 1.8
pPR8	6/20 (30)	32.9 ± 9.1	8.2 ± 1.2
Control	0/20 (0)	34.1 ± 4.3	9.2 ± 1.2

*^a^Female BALB/c mice (6–8 weeks old) were immunized twice, at an interval of 3 weeks, with 30 µg of plasmid DNAs substituted with two epitopes of the Pdm09 HA. 2 weeks after the boost, the mice were challenged with a lethal dose (20 × MLD_50_) of mouse-adapted Pdm09 virus and monitored for body weight loss and survival for 14 days (*n* = 20). The BAL was collected 4 days post-infection for titration of lung virus (*n* = 6)*.

*^b^Results are expressed as the means ± SD of each group*.

*^c^Significant difference compared with pPR8 (*p* < 0.05)*.

**Figure 5:**
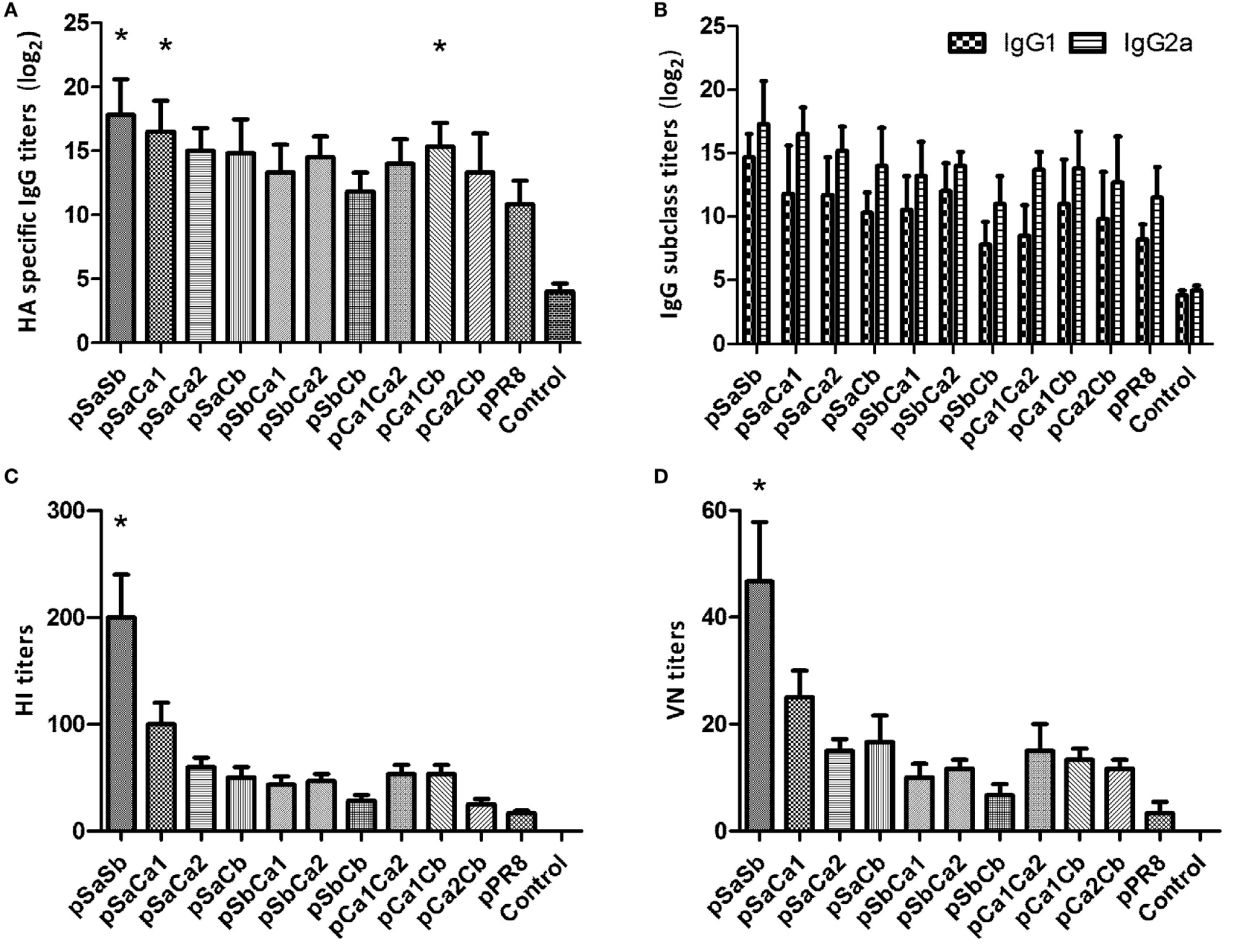
Antibody responses in mice immunized with plasmid DNAs substituted with two epitopes of the Pdm09 HA. Female BALB/c mice (6–8 weeks old) were immunized twice, at an interval of 3 weeks, with 30 µg of plasmid DNAs substituted with two epitopes of the Pdm09 HA. Thirteen days after the boost, the sera were collected for Ab analyses; **(A)** for immunoglobulin G (IgG); **(B)** for IgG subclass; **(C)** for hemagglutination-inhibition assay; and **(D)** for VN assay. Bars and whiskers represent the average and SD. Asterisk symbol (*) indicates a significant difference compared with pPR8 (*p* < 0.05).

We next analyzed the levels of IgG, IgG subclass, HI, and VN antibody titers in each group. As shown in Figure 5, mice immunized with pSaSb induced the highest level of Abs, while pSbCb was the least effective in eliciting an antibody response; nevertheless, the levels of IgG Abs in all groups were higher than those in the pPR8 group, with pSaSb, pSaCa1, and pCa1Cb being statistically significant (*p* < 0.05). These data were consistent with the survival rate and body weight data. Notably, while mice immunized with plasmid DNAs containing two epitopes generally displayed increased HI and VN antibody levels compared with pPR8, the increased antibody levels were only statistically significant in mice immunized with pSaSb. With respect to the IgG subclass, the levels detected after the immunization of plasmid DNAs with two epitopes were the same as those obtained with one-epitope constructs in terms of the ratio between IgG1 and IgG2a.

Notably, the combination of Sa and Sb provided complete protection, while other combinations were much less effective. Specifically, while pSaCa1, pSaCa2, pSbCa2, pCa1Ca2, and pCa1Cb could induce much better protection than their respective single-epitope constructs, some combinations of dual epitopes, such as pSaCb, induced protection similar to that induced by single-epitope plasmid DNAs following lethal viral challenge.

## Discussion

The results of the present study revealed that better protection against lethal dose pandemic influenza virus challenge could be achieved by merely swapping a single epitope in the HA proteins derived from different viral strains and that the substitution of two epitopes could substantially enhance protection. To the best of our knowledge, these findings have not previously been reported.

Systemic analyses of epitopes in HA using neutralizing mAbs have revealed that the HA proteins of both H1 and H3 subtypes contain five major antigenic sites (10, 11, 28, 29). Epitopes Sa and Sb in H1 correspond to sites A and B in H3, while Ca1 and Ca2 in H1 share a large overlapping region with site D in H3, and Cb in H1 is similar to E in H3 and located around the bottom of the HA head. In human influenza virus, A and B sites are major neutralizing epitopes of H3 HA (30–32). However, in the present study, DNA vaccines containing epitopes Sa and Ca1 (homologous to sites A and D in H3, respectively) induced better protection. This discrepancy may reflect differences in the experimental conditions and/or dominant epitopes. Moreover, studies on H7N9 revealed a relatively conservative epitope corresponding to site A in H3, and the passive transfer of monoclonal Abs could protect naïve mice against viral challenge (33).

Recently, Yewdell et al. investigated the immunodominance of five HA globular domain antigenic sites in PR8 HA through the selection of a panel of PR8 escape mutants that maintain antigenicity in one of five antigenic sites (34). These authors observed that the hierarchy of serum antibody titers specific for each epitope was dependent on the antigen formulation, the route of administration and the genetic background of the animals. The Sa-specific Abs demonstrated relatively higher HI and VN activities, suggesting that epitope proximity to the sialic acid receptor site dictated *in vitro* neutralization activities. The findings in this study were largely consistent with the results of the present study, i.e., epitope Sa provided better protection against lethal virus challenge and induced higher strain-specific antibody titers.

Previous epitope mapping experiments with Pdm09 HA revealed that the epitopes of most monoclonal Abs were located in Sa, Sb, and Ca2, suggesting that these epitopes were markedly immunogenic (35). According to the epidemiological surveillance after the pandemic, a slight antigenic drift was observed for the Pdm09 virus, there were some amino acid mutations in its neutralizing epitopes, which may affect viral pathogenesis. For example, a prevalent mutation D239G in the Ca2 epitope in the early phase of the H1N1 pandemic was associated with severe clinical symptoms (36); an N142D mutation in the Sa epitope in viral isolates, most of which were coupled with E391K, was observed in the Southern Hemisphere in 2010 (37). Importantly, subsequent monitoring confirmed that the newly emerged double mutation N142D/N173K in the Sa epitope markedly reduces antibody binding to the Pdm09 virus (38). These mutations revealed that selective immune pressures could impact these epitopes. These observations and those in the present study suggest that monitoring these epitopes is of crucial importance.

Interestingly, mice immunized with pPR8 in the present study also had partial protection against lethal infection of the Pdm09 virus, although the amino acid sequences of the neutralizing epitopes are markedly different. This protection might partly reflect T cell responses. Indeed, previous studies have demonstrated that intramuscular immunization followed by electroporation could induce a Th1-skewed response and could increase the proliferation of virus-specific CTLs (39–41). It is also possible that the protection afforded by pPR8 could be mediated through Abs against other parts of HA. Specifically, numerous studies have shown that the immune response against the HA stem can provide cross-protection against influenza virus infection (42–45).

In the two-epitope combination experiments, we observed that several combinations of two epitopes, i.e., pSaSb and pSaCa1, pSaCa2 and pSbCa2, and pCa1Ca2 and pCa1Cb, showed remarkably increased protection compared with their corresponding single epitope-substituted groups. Examination of the spatial location of these epitopes in the crystal structures revealed that epitopes Sa and Sb are closest to each other, followed by Sb and Ca2. Epitopes Sa and Ca1 are also close to each other in the monomer, whereas the Ca1 and Ca2 are adjacent to each other in the trimer of HAs. Further studies are required to determine whether enhanced antibody binding reflects the fact that the two epitopes are close to each other in the HA proteins. Notably, recent studies using monoclonal Abs against Pdm09 virus HA have revealed various neutralizing Abs that recognize both Sa and Sb sites in addition to direct interactions with receptor-binding sites (46, 47); it was also suggested that dividing HA antigens into five neutralizing sites might be oversimplified (35, 48, 49). Regardless, the results of the present study support the notion that multiple combinations of epitopes should be considered to improve the protective efficacy.

Overall, in the present study, we analyzed the protective immune responses provided by HA epitopes, which could contribute to the rational design and optimization of new influenza vaccines and to the screening of future vaccine strains.

## Ethics Statement

Specific pathogen-free (SPF) female BALB/c mice (6–8 weeks old) were purchased from the Center for Disease Control and Prevention of Hubei Province and were housed under SPF conditions with constant temperature and humidity. All mice had free access to food and water. All experiments with mice were reviewed and approved by the Institutional Animal Care and Use Committee of Hunan Normal University. All animal procedures were performed in accordance with the animal ethics guidelines of the Chinese National Health and Medical Research Council (NHMRC).

## Author Contributions

BP and ZC conceived and designed the experiments. BP performed the experiments and analyzed the data. NP, YZ, FZ, HC, FF, FW, and FL contributed conceptual advice and technical support. BP, XL, and ZC drafted the manuscript.

## Conflict of Interest Statement

The authors declare that the research was conducted in the absence of any commercial or financial relationships that could be construed as a potential conflict of interest. The reviewer, FB, and handling editor declared their shared affiliation.

## Supplementary Material

Figure S1 Immunofluorescence (IF) images of the expressed plasmid DNAs. The plasmid DNAs encoding viral HA protein or empty vector were expressed in 293T cells. IF staining was performed with PR8 HA-specific mouse antiserum, followed by a secondary Alexa Fluor488-labeled goat anti-mouse antibody. Images are shown in parallel in IF and white light.

Figure S2 Western blotting of the expressed plasmid DNAs. Cell lysates obtained from 293T cells transfected with the plasmid DNAs encoding viral HA protein or empty vector were subjected to SDS-PAGE. Protein bands were visualized using PR8 HA specific mouse antiserum or an anti-actin monoclonal antibody, followed by a horseradish peroxidase-conjugated goat anti-mouse antibody.

Figure S3 Hemadsorption of expressed plasmid DNAs. The 293T cells were transfected with the plasmid DNAs encoding viral HA protein or empty vector. 24 h later, the cells were incubated with cRBC to examine for hemadsorption by the expressed HAs.
