# Supplementary material for: Comparison of the Protective Efficacy of Neutralizing Epitopes of 2009 Pandemic H1N1 Influenza Hemagglutinin
Source: Front Immunol. 2017 Aug 31;8:1070. doi: 10.3389/fimmu.2017.01070 (PMC5583165; doi:10.3389/fimmu.2017.01070)
Supplement: Supplementary file 1 [file Data_Sheet_1.DOCX]

**Supplementary Materials and Methods**

**Immunofluorescence (IF)**

293T cells were transfected with 0.25 μg of plasmid DNAs encoding viral HA protein or empty vector using Lipofectamine 2000 (Invitrogen) in serum-free medium on 24-well tissue culture plates. After incubation for 24 h, the cells were washed with PBS. Immunofluorescence staining was performed with PR8-HA-specific mouse antiserum (polyclonal antibody), followed by a secondary Alexa Fluor488-labelled goat anti-mouse antibody. Fluorescence imaging was performed with a fluorescence microscope (Olympus IX71, Japan).

**Western Blotting**

The 293T cells were transfected with 1 μg of plasmid DNAs encoding viral HA protein or empty vector as described above on 6-well tissue culture plates. After incubation for 48 h, the transfected cells were washed three times with PBS and subsequently [cell lysis](http://www.baidu.com/link?url=fM0_-_Nz8kf8EWmXxnTDZ6CnRWmi6OwWWZUcdFEonxxsbsUPd7kzfsP1e7jrcf4qcplvqcFJ7dEPVtdhilzpQ_F6gUZia8Cv-tr10ksZPQ_Htop_8TSWosnbZq7TAnxA) solution was added. Thirty micrograms of total protein from the cells was electrophoresed on a 10% Tris-glycine gel and transferred onto a PVDF membrane. The membrane was blocked in 5% fat-free milk and incubated with PR8-HA specific mouse antiserum (polyclonal antibody) or an anti-actin monoclonal antibody, followed by incubation with horseradish peroxidase (HRP) conjugated goat anti-mouse IgG antibody. Protein bands were quantified using Molecular Imager chemiDoc^TM^ XRS+ with Image Lab^TM^ software (BioRad, USA). The expression levels of the HAs were normalized to actin.

**Haemadsorption**
The 293T cells were transfected with 0.25 μg of plasmid DNAs encoding viral HA protein or empty vector as above on 24-well tissue culture plate. After incubation for 24 h, the transfected cells were treated with 10 mU of neuraminidase at 37°C for 60 min and subsequently incubated with 3% chicken red blood cells (cRBC) for 30 min. After a thorough wash with PBS, the cells were examined for haemadsorption with phase-contrast microscopy using an IX71 inverted microscope with a 40X objective (Olympus, Japan).
