# Supplementary figures and images for: Comparison of the Protective Efficacy of Neutralizing Epitopes of 2009 Pandemic H1N1 Influenza Hemagglutinin
Source: Front Immunol. 2017 Aug 31;8:1070. doi: 10.3389/fimmu.2017.01070 (PMC5583165; doi:10.3389/fimmu.2017.01070)

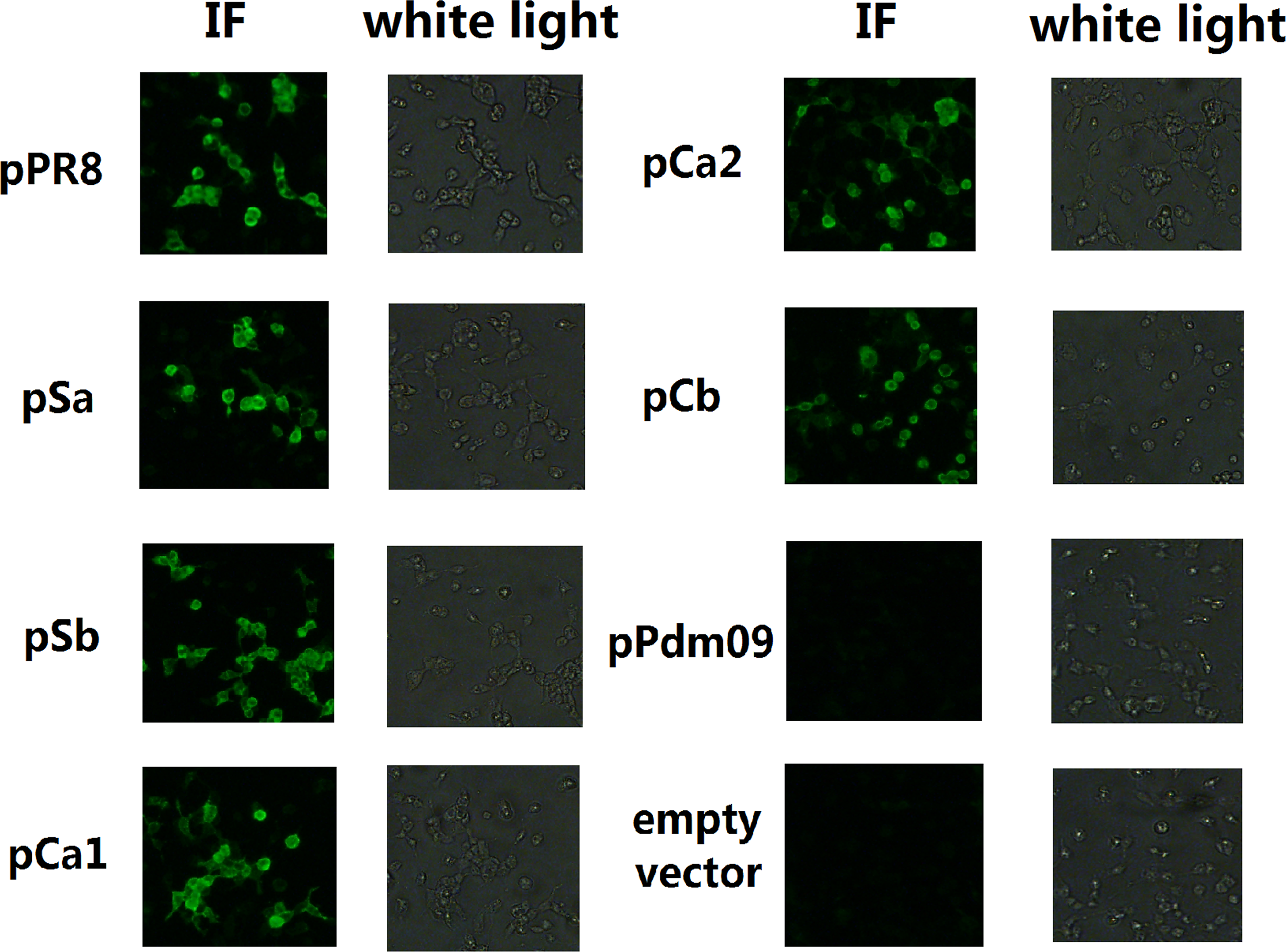

Supplement: Figure S1 — Immunofluorescence (IF) images of the expressed plasmid DNAs. The plasmid DNAs encoding viral HA protein or empty vector were expressed in 293T cells. IF staining was performed with PR8 HA-specific mouse antiserum, followed by a secondary Alexa Fluor488-labeled goat anti-mouse antibody. Images are shown in parallel in IF and white light. [file Image_1.TIF]

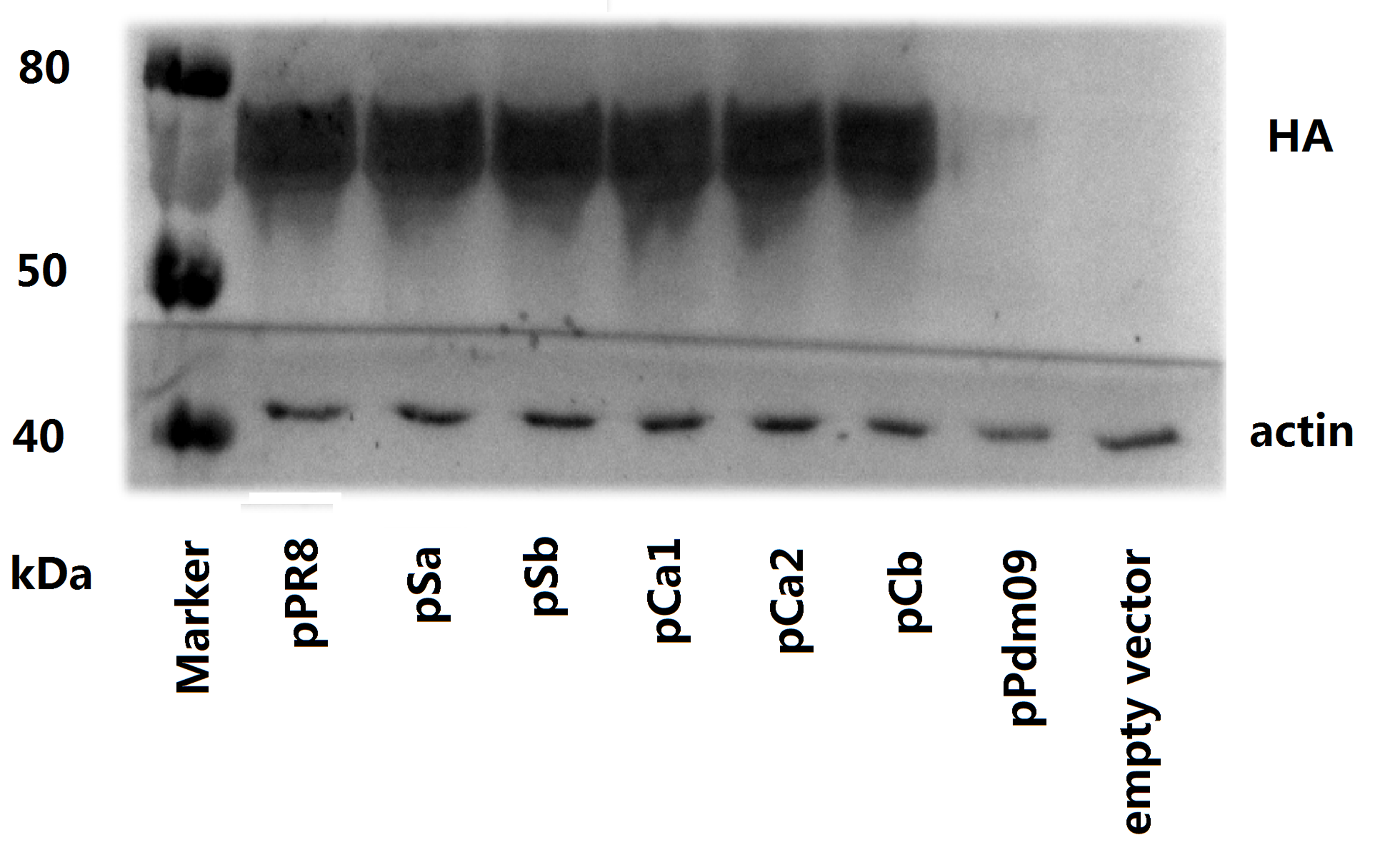

Supplement: Figure S2 — Western blotting of the expressed plasmid DNAs. Cell lysates obtained from 293T cells transfected with the plasmid DNAs encoding viral HA protein or empty vector were subjected to SDS-PAGE. Protein bands were visualized using PR8 HA specific mouse antiserum or an anti-actin monoclonal antibody, followed by a horseradish peroxidase-conjugated goat anti-mouse antibody. [file Image_2.TIF]

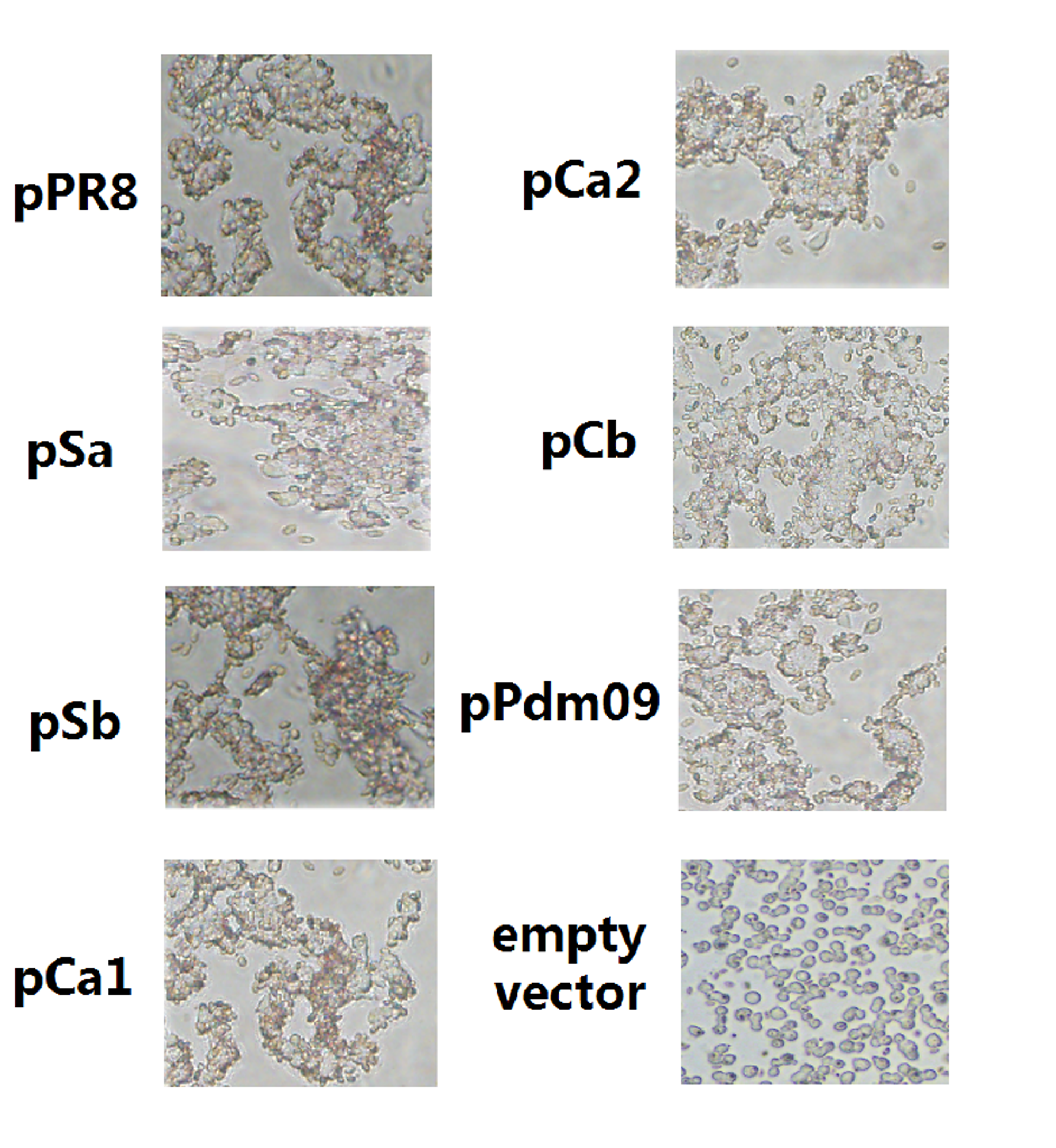

Supplement: Figure S3 — Hemadsorption of expressed plasmid DNAs. The 293T cells were transfected with the plasmid DNAs encoding viral HA protein or empty vector. 24 h later, the cells were incubated with cRBC to examine for hemadsorption by the expressed HAs. [file Image_3.TIF]
